# Digital interventions for people with dementia and carers: effective, cost-effective and equitable?

**DOI:** 10.2217/nmt-2022-0025

**Published:** 2022-07-14

**Authors:** Martin Knapp, Xheni Shehaj, Gloria Wong

**Affiliations:** 1 Care Policy & Evaluation Centre, London School of Economics & Political Science, London, UK; 2 Department of Social Work & Social Administration, The University of Hong Kong, Hong Kong SAR, China; 3 Department of Clinical, Educational & Health Psychology, University College London, London, UK; 4 Department of Health Service & Population Research, Institute of Psychiatry, Psychology & Neuroscience (IoPPN), King’s College London, London, UK

**Keywords:** access, assistive technology, cost, cost–effectiveness, digital technology, information and communication technologies, mobile technology, quality-of-life, technology readiness, telecare

## Opportunity for a ‘digital revolution’

As the number of people living with dementia continues to grow [1], the need to plan a response to this global public health challenge – indeed, global, social and economic challenge – is obvious. Enormous scientific efforts are rightly focused on finding a disease-modifying treatment (‘cure’ in the public vernacular), and there is also increasing emphasis on risk-reduction through changes in lifestyle and public health efforts across the life-course.

However, breakthroughs in understanding how to alter the underlying pathophysiological mechanisms of the disease will not make a difference to the 57 million people who currently live with dementia. Nor will identification of another risk factor or success in changing those midlife behaviors or circumstances that exacerbate it. Indeed, it is highly likely that almost no one who develops dementia over the next decade will feel any direct benefits themselves from new discoveries in basic science or galvanized public health measures. Any such changes will take rather longer to bed down.

This is not in any way to downplay the importance of such efforts but is simply to recognize the enormously complex science needed to make disease-modifying discoveries, and the equally large implementation tasks to achieve change early enough in the disease course to make a difference. There are equivalent implementation barriers facing the best public health measures – those that require widespread societal and individual behavior changes to change the epidemiology of dementia. The perceived affordability of such changes is probably an issue too.

These are the reasons why governments across the world must take action now to support people living with dementia and their family or other caregivers. Good-quality care and support interventions are urgently needed to improve quality-of-life (QoL) and ensure universal coverage of (at least) basic care for everyone already living with dementia [2].

Since the beginnings of time, care for someone with a need has been inter-personal – one person supporting another, perhaps with reciprocation – and, in the context of dementia, the quality of that care has pivoted on the quality of the one-to-one relationship. Humans learned very early to use tools, of course, and ‘technology’ of various basic and more sophisticated kinds has therefore long been used to assist people who may struggle with the activities of daily living: wheelchairs, grabrails, hearing aids, wet-rooms and continence pads are obvious examples.

But what we are seeing now is widespread recognition – and certainly widespread hope – that digital technology could be a game-changer. The relevance of such technology is now greater than most of us previously realized. One reason is obviously the growing dementia prevalence noted above. Much of the huge demand for established care and support interventions – which require workforce to deliver – is unmet, with shortages of care workers a major challenge across most of the world. There are other challenges, such as geographical barriers and mobility issues that constrain access to in-person services. Thus, even though scaling up of existing evidence-based interventions such as cognitive stimulation therapy is in theory an affordable, cost-effective way to improve lives of people with dementia [3], accessing these interventions will likely be hugely difficult.

Another factor is experience accumulated during the COVID-19 pandemic. With restrictions on visitors to and visits out of congregate care settings, for example, technology replaced in-person contact as an infection-control strategy, such as the use of electronic devices to connect care home residents with their families [4]. National lockdowns also forced many services to move from in-person to online, including shopping, education and some healthcare. The pandemic became a natural experiment of feasibility: many people were cajoled into using digital technology, discovering that – even with some cognitive impairment and low information and communication technology (ICT) literacy – these forms of communication can be managed and can be helpful.

These new experiences might generally have been relatively straightforward, such as online grocery orders, consultations with primary care staff or family gatherings on Zoom, but they auger well for the future development of more complex digital engagement. For example, new attempts are being made to support use of ICT to deliver cognitive stimulation therapy and carer support [5], with initial evidence on feasibility to support its recommendation for people unable to access traditional in-person services [6]. There is, more generally, a common sentiment that a ‘digital revolution’ in dementia care is necessary [7], and that telecare has a central role for future dementia care [8].

## A repeating story

This is the context in which the Department of Health and Social Care in England commissioned our rapid review to understand the current evidence and readiness of digital technologies in supporting people living with dementia and carers to live well [9]. While there have been many published reports in the last two decades of digital technologies in the dementia field, and probably thousands of such technologies being developed right now, there is still rather sparse evidence of effectiveness in improving health, independent living or QoL. And there is almost no evidence as to whether these technologies would be considered cost-effective by funding or health technology assessment bodies.

Based on the EU Horizon 2020 Technology Readiness Level framework [10], technologies that appear most ready for use in the next 5 years – in other words, no longer in ideas or prototype stage include: virtual care for tailored strategies for carers; mobile technologies for supporting self-care and daily activities; touchscreen and multimedia interventions and activities (such as digital life storybook) to improve mood, engagement and behaviors; and ICT-based technologies for social connection.

The general quality of evidence on effectiveness is low, with only a few exceptions. For most digital technologies, we do not know whether they address dementia symptoms, improve other aspects of health, promote independence in daily living, reduce care staff time, reduce the time that family or other unpaid carers spend providing support or enhance the subjective wellbeing of people with the condition or those carers. In reaching this conclusion, our rapid review reaffirmed what others had found previously [11].

It is hardly surprising that economic evidence on digital technologies was even more sparse: there has been hardly any new good-quality cost–effectiveness evidence since a review of assistive technology almost a decade ago [12]. The few studies in recent years that have included a cost component, have been hamstrung by methodological limitations: no control groups, incomplete cost information, lack of outcome data [9]. The good news is that we found several protocols for economic evaluations, which hopefully will soon provide some robust insights.

It is perhaps just as important to note negative findings where some of the more ready technologies have been evaluated. In a well-designed randomized controlled trial (Assistive Technology and Telecare to maintain Independent Living At home in people with dementia, [ATTILA]) to examine the effectiveness and cost–effectiveness of assistive technology and telecare, it was found that the ATTILA intervention (a full package of assistive technology and telecare including reminder/prompting, safety, communication, support leisure time and other devices) did not produce any better outcomes when compared with smoke and carbon monoxide detectors and a pendant alarm; there was no effect on time to nursing home admission, carer burden or psychological wellbeing [13], while there may even be some reduction in quality-adjusted life years [14]. Limited fidelity of technology recommendation to the person’s needs may be the reason, which is a common issue. Such findings echo those from a linked rapid review on fall prevention technologies in dementia [15], in which the fact that some alarm systems can be ‘unsettling’ for people living with dementia, despite their potentials in reducing fall risk.

## What needs to be revolutionized

If we are to achieve the much-heralded ‘digital revolution’ in care, and particularly to build on the few positive experiences during the pandemic, then we first need to remind ourselves how little progress has been made to date. The root problem is a repeating story: enthusiasm in invention and innovation that sometimes translates into production or even validation, but rarely moves on to widespread real-world application because of an absence of convincing evidence of effectiveness.

Looking at the more successful digital technologies for dementia, a common feature appears to be that they were designed to achieve outcomes that align with core dementia care principles, namely person-centered care that emphasizes social connectedness and meaningful engagement. By the same token, digital technologies that focus on what might be called lower-level human needs such as safety and physical health, are less likely to be effective when evaluated in terms of commonly valued criteria such as QoL, psychological wellbeing and value for money.

Another key feature of the more promising digital technologies in the dementia area is their use of existing commercial solutions instead of specifically developing technologies solely for dementia care and support. This should not be surprising: the available evidence on technological innovations in dementia contexts mirrors what has been observed in other areas of health and social care, for example as conceptualized and analyzed by Greenhalgh *et al.,* in their NASSS framework: non adoption, abandonment, difficulties in scaling up, spread and sustainability [16].

The deployment or repurposing of existing commercial solutions has been more successful simply because many barriers to scaling up have already been overcome, including trust (so important in older populations), design (including to address loss of sensory acuity), price (benefitting from economies of scale) and general awareness. If future innovations pay enough attention not only to effectiveness and cost–effectiveness, but also to scaling-up barriers (which could be linked), more substantial progress could be possible, given the current level of interest and research in digital technologies for dementia care and interventions.

Lessons learned from the slow progress in this field probably boil down to the fundamental issue of how to position digital technologies in the overall development of dementia care and support. When set in that wider context of advancing non-pharmacological care and intervention, with digital technology being just one delivery mode that may supplement or complement others, the repeating story of enthusiasm and efforts ending nowhere may finally find a new chapter – one that promotes equity in accessing affordable, high-quality care.

## Concerted innovation efforts for equity of access

There are effective and cost-effective interventions and support arrangements already available in the ‘non-digital world’ (see 17 for a summary of evidence). Access to these interventions and support; however, is often limited by workforce shortages or logistical challenges. Digital technology could offer a solution. Access to health and social care is multidimensional, depending on how care services interface with populations. It could be operationalized as the degree of fit along each of a number of dimensions: approachability, acceptability, availability and accommodation, affordability and appropriateness [18].

Care and interventions delivered using digital technology may be able to overcome some of the access barriers, such as limited mobility of some frail older persons, travel distances for people in remote communities, shortages of staff skilled in the provision of in-person therapies. On the other hand, of course, digital technologies bring their own barriers: poor IT literacy; infrastructural support; fears about digital fraud and privacy; unaffordability of equipment or data charges; and poor operability especially when sensory skills decline, to name a few. Some of these barriers are also linked to socioeconomic disadvantage, and some might be greater in some minority population groups (for language or cultural reasons). The COVID pandemic provided an opportunity to address some of the inequities. An example is the UN International Telecommunication Union’s Connect2Recovery initiative to ensure affordable and reliable connectivity in countries needing support (www.itu.int/en/ITU-D/Pages/connect-2-recover.aspx), starting with supporting Africa, to create ‘an open standard for terrestrial optical fiber cable infrastructure data’ and a digital map of the infrastructure for all.

Digital and ‘offline’ approaches have their respective advantages when seeking to improve access to dementia care and interventions. For example, in a US study of a video-based intervention for family carers to receive tailored advice on care strategies (FamTechCare), the digital (video) support improved carer outcomes when compared with telephone-based support, probably due to better tailoring. However, when used with people with more severe dementia, the video-based intervention may be less acceptable due to privacy concerns [19]. Other more obvious considerations in improving access would include the ability of digital technologies to reach much larger audiences (e.g., online psychoeducation) in circumstances where low-intensity, less interactive communication is appropriate for the intervention. In contrast, intervention and care which is more communication- or interaction-intensive (e.g., group therapy), traditional in-person sessions may be more facilitative for larger groups.

These differences and comparative advantages raise important questions and offer potential solutions. A mixed portfolio of digital and non-digital approaches will increase the likelihood of achieving equitable access to care arrangements and therapeutic interventions that are effective and cost-effective.

## A way forward: the best of both worlds?

Much of the dialogue in discussions of, and concerns about digital technologies in dementia care has implicitly assumed that these are standalone strategies that either replace or compete with traditional services. If funding bodies or policymakers take such a stance, then it is absolutely legitimate to worry about people with lower ICT literacy or from lower-resource settings being further disadvantaged in access to services delivered only through digital platforms (e.g., smart phones or the internet). But this does not need to be the case. Researchers and advocacy bodies need to be clear in their dealings with service commissioners and decision-makers that digital technologies should be seen as options or enrichments, not alternatives, to existing evidence-based care and interventions. That is the way to make the biggest improvements in QoL for people living with dementia and their carers.

For many dementia services we should aspire to ‘the best of both worlds’: hybrid mode of services (e.g., having some sessions in-person and some online), offering choice of digital or in-person care and intervention (e.g., based on preference, affordability, availability of infrastructural support, and certainly informed by evidence) and use of digital technologies to improve certain aspects of traditional services (e.g., using smart technologies to facilitate professional care delivered in-person by humans). Such arrangement would not be novel in the wider health and social care context, but we have yet to get the best out of them to improve dementia care and interventions.

To release the potential that digital technologies obviously hold, a focus on key outcomes, namely QoL and wellbeing – rather than an emphasis on format, which can be digital, non digital, or mixed – would be the best strategy. Innovations that make a real difference to more people’s QoL are, by their very nature, more likely to be effective and cost-effective when evaluated against outcomes that matter. There would then be greater justification – from resource and equity perspectives – for them to be prioritized, adopted and scaled up.

New developments in digital technologies should be incentivized and steered toward outcomes that matter for people living with dementia and their carers. By reminding ourselves of Maslow’s law of the instrument – ‘If the only tool you have is a hammer, it is tempting to treat everything as if it were a nail’ [20] – we could break out of the repeating pattern of focusing too much on the method and forgetting the purpose, and thereby allow a real digital revolution to happen in dementia care.
